# Epidemiology of Syphilis in Pregnancy and Congenital Syphilis in Brazil and the Risk or Associated Factors: Protocol for a Systematic Review

**DOI:** 10.2196/50702

**Published:** 2024-01-04

**Authors:** Yago Tavares Pinheiro, Janmilli da Costa Dantas, Jose Rebberty Rodrigo Holanda, Ankilma do Nascimento Andrade Feitosa, Richardson Augusto Rosendo da Silva

**Affiliations:** 1 Federal University of Rio Grande do Norte Natal Brazil; 2 Federal University of Rio Grande do Norte Caicó Brazil; 3 University Center Santa Maria Cajazeiras Brazil

**Keywords:** sexually transmitted diseases, epidemiology, prevalence, incidence, Brazil, syphilis, pregnancy, sociocultural, economic, congenital syphilis, heterogeneity, decision-making

## Abstract

**Background:**

Syphilis in pregnancy and congenital syphilis are growing public health issues worldwide. Several factors can influence their occurrence in the population. Therefore, understanding the epidemiology of this condition and the factors that influence its occurrence is fundamental for decision-making by clinicians and health managers. However, so far, no systematic review has summarized and analyzed data on the incidence, prevalence, and predictors of these diseases in Brazilian cities, considering different sociocultural, demographic, economic, sanitary, and spatial-temporal characteristics presented across locations.

**Objective:**

We propose a systematic review protocol to gather and analyze data on the incidence, prevalence, and risk or associated factors of syphilis in pregnancy and congenital syphilis in Brazil, taking into account different local or regional contexts.

**Methods:**

Searches will be conducted in CINAHL, MEDLINE, LILACS, Embase, and Web of Science databases. We will include observational studies (ie, cross-sectional, longitudinal, or case-control studies), analyzing the incidence, prevalence, and risk or associated factors of syphilis in pregnancy and congenital syphilis in Brazil from primary data. The diagnosed syphilis will be assessed based on direct pathogen detection tests or through immunological, treponemal or nontreponemal tests, following Brazilian protocols for diagnosing syphilis. The studies are currently undergoing screening in the databases, and after this step, 2 reviewers will perform all identified documents. The Newcastle-Ottawa Scale and the GRADE (Grading of Recommendations, Assessment, Development, and Evaluations) system will be used to assess methodological quality and quality of evidence of studies, respectively. The Kappa coefficient will assess the agreement between researchers in each study stage. Cochran Q test will assess the heterogeneity among studies. Then, a random-effects meta-analysis will be performed.

**Results:**

Results will be discussed based on subgroup analysis, which is as follows: (1) type of syphilis (in pregnancy or congenital), (2) type of study (case-control and cross-sectional studies for analysis of associated factors and longitudinal studies for risk factors), and (3) contextual factors (ie, region of country, socioeconomic and demographic characteristics, and year of study). This systematic review is expected to be completed by December 2023, and our results will be disseminated through publication in peer-reviewed journals and scientific events.

**Conclusions:**

This systematic review aims to assist health care managers and professionals in their decision-making to control these diseases in Brazil, considering location heterogeneity. Furthermore, countries with health systems and demographic and socioeconomic contexts similar to those of Brazil may benefit from this information.

**International Registered Report Identifier (IRRID):**

DERR1-10.2196/50702

## Introduction

Syphilis is a sexually transmitted infection caused by *Treponema pallidum* and is a major public health issue worldwide [1]. Despite the efforts of health care professionals to control this infection in Brazil, cases of syphilis have increased in recent years [2-4], impacting public and private health care systems and highlighting the need to improve disease surveillance [5,6]. Globally, 2 million out of 36 million syphilis infections occur in pregnant women [7], resulting in congenital syphilis (infection of the fetus) and adverse events (eg, early fetal death, stillbirth, premature birth, low birth weight, and neonatal death) [8,9].

Recently, an outbreak of syphilis has been observed among men and women in more economically developed countries, which can be explained by changes in the sexual behavior of individuals and increased exposure to the risk of infection due to a false sense of security stemming from new treatments and an increased search for sexual partners over the internet [10]. In this context, understanding the epidemiology and control of this disease becomes more complex and difficult.

Syphilis in pregnancy and congenital syphilis can be controlled with health care measures, such as access to prevention services, early diagnosis, and treatment [4,11,12]. Conversely, these measures require the analysis of epidemiological data and predictors [4,11]. Although studies in Brazilian cities analyzed the incidence, prevalence, and predictors of syphilis in pregnancy and congenital syphilis [13-17], each city presented different sociocultural, demographic, economic, sanitary, and spatial-temporal characteristics, hindering data extrapolation to the national territory.

Summarizing and analyzing the incidence, prevalence, and risk or associated factors of syphilis in pregnancy and congenital syphilis must take into account location heterogeneity. This type of analysis enables a broader understanding of the problem, improves control strategies and equity in disease management, and establishes reference data to help disease screening efforts in Brazil. Despite the relevance of the theme, no systematic review has been conducted to date on the epidemiological data of gestational and congenital syphilis or its predictors, subgrouping and analyzing this information from different contexts.

Thus, we propose a systematic review protocol to gather and analyze data on the incidence, prevalence, and risk or associated factors of syphilis in pregnancy and congenital syphilis in Brazil, taking into account different local or regional contexts.

## Methods

### Study Design

This systematic review protocol was developed according to PRISMA-P (Preferred Reporting Items for Systematic Review and Meta-Analysis Protocols) [18], which will also guide the systematic review. The protocol has been registered in PROSPERO (CRD42022329329).

### Eligibility Criteria

Observational studies whose sample comprised cases of syphilis in pregnant women or newborns in Brazil will be included in the systematic review. Table 1 [2,19,20] presents the eligibility criteria used in the review.

**Table 1:** Eligibility criteria for the systematic review.

Variables	Inclusion criteria	Exclusion criteria
Study design	Cross-sectional, longitudinal, or case-control studies conducted in Brazil Studies based on primary data	Reviews, opinion articles, editorials, or publications without primary data or not peer-reviewed The most complete and recent data will be used if studies report the same data in multiple sources [19,20].
Population and location	Studies in which the sample involved pregnant women or newborns with syphilis Studies with residents in Brazil	Studies with samples involving other populations (nonpregnant women or men)
Outcomes	Studies reporting the prevalence or incidence of Treponema pallidum (syphilis) infection or its risk or associated factors in pregnant women or neonates Studies that diagnosed syphilis based on direct pathogen detection tests or through immunological, treponemal, or nontreponemal tests, in accordance with Brazilian protocols for diagnosing syphilis [2]	Studies presenting the incidence or prevalence of combined infections (ie, syphilis with other sexually transmitted infections) and not allowing isolated analysis
Language	—^a^	No restriction regarding language or year of publication
Time frame	—	No restriction regarding to year in which the study was carried out or published

^a^Not applicable.

### Study Selection

Textbox 1 presents the search strategy. Searches will be conducted in the CINAHL, MEDLINE, LILACS, Embase, and Web of Science databases. Grey literature will be also searched using the reference lists of relevant studies in addition to using databases such as Open Gray and Google Scholar. Further, reports with epidemiological data on syphilis in the country will be screened in the electronic database of the Brazilian Ministry of Health.

Two researchers will independently search, identify potentially eligible studies, and remove duplicates. Then, inclusion and exclusion criteria will be applied to titles and abstracts, eligible studies will be read in full, and reasons for exclusion will be recorded. Disagreements between researchers will be resolved by discussion or with a third researcher. The flowchart of the study selection is described in Figure 1.

**Search strategy.**

#1 “syphilis” [Title/Abstract] OR “congenital syphilis” [Title/Abstract] OR “treponemal infections” [Title/Abstract] OR “T. pallidum” [Title/Abstract] OR “pallidum” [Title/Abstract] OR “serosyphilis” [Title/Abstract] OR “sexually transmitted diseases” [Title/Abstract]

#2 “pregnant” [Title/Abstract] OR “women” [Title/Abstract] OR “congenital” [Title/Abstract]

#3 “incidence” [Title/Abstract] OR “prevalence” [Title/Abstract] OR “prevalence study” [Title/Abstract] OR “cross-sectional study” [Title/Abstract] OR “observational study” [Title/Abstract]

#4 “risk factors” [Title/Abstract] OR “associated factors” [Title/Abstract] OR “measures of association, exposure, risk or outcome” [Title/Abstract]

#5 “brazil” [Title/Abstract] OR “brazilian” [Title/Abstract]

#6 #1 AND #2 AND #3 OR #4 AND #5

**Figure 1:**
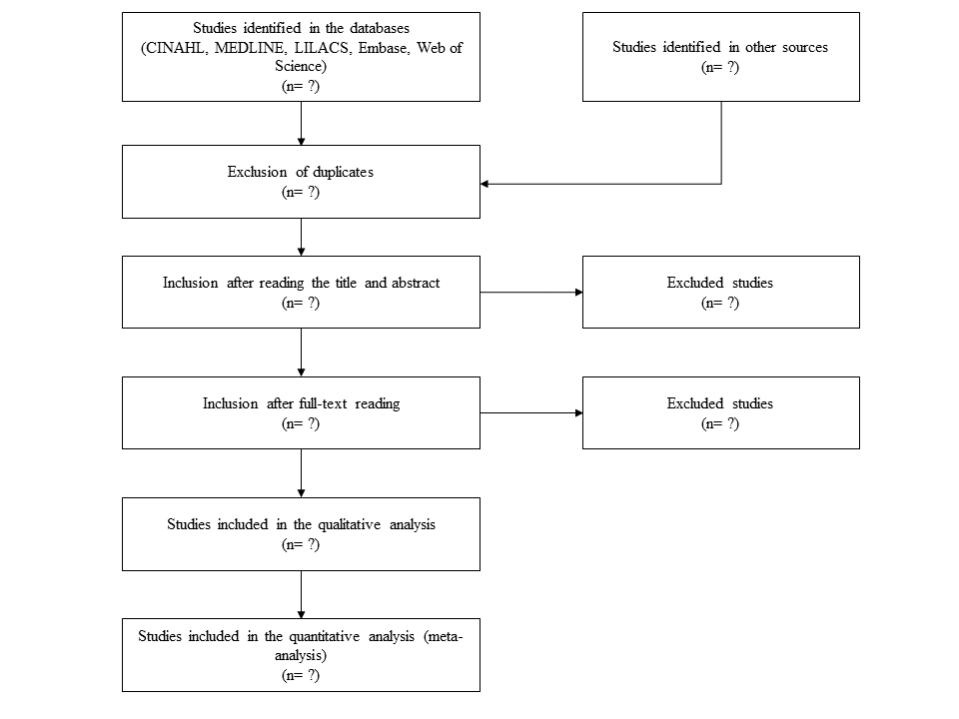
Flowchart of the study. The numbers are to be confirmed.

### Assessment of Methodological Quality and Quality of Evidence

The Newcastle-Ottawa Scale will be used to assess the methodological quality of studies [21]. This scale includes 8 items categorized into 3 domains (ie, selection, comparability, and outcome or exposure) to assess the risk of bias in nonrandomized studies. The Newcastle-Ottawa Scale has specific tools for cohort and case-control studies. Thus, adaptations will be made to allow the proper assessment of the potential sources of bias in cross-sectional studies. In addition, quality of evidence will be analyzed using the GRADE (Grading of Recommendations, Assessment, Development, and Evaluations) system, which classifies evidence as high, moderate, low, and very low [22,23].

### Data Extraction and Synthesis

The following data will be extracted and entered into a Microsoft Excel spreadsheet: first author, year of publication, type of study (eg, longitudinal, cross-sectional, or case-control), type of syphilis (eg, in pregnancy or congenital), the diagnostic method used, study location (eg, city and state), participants (eg, sample size, age, type of population, and presence of co-infection), population setting (eg, community, health care centers, schools, neighborhoods, and the environmental context of the participants), date of data collection, sampling method, and main results (eg, incidence, prevalence, and risk or associated factors).

After summarizing the studies, results will be discussed based on subgroup analysis, as follows: (1) type of syphilis (eg, in pregnancy or congenital), (2) type of study (eg, case-control and cross-sectional for analysis of associated factors and longitudinal for risk factors), and (3) contextual factors (eg, region of country, socioeconomic and demographic characteristics, and year of study).

### Statistical Planning

Kappa coefficient will assess the agreement between researchers [24]. The unadjusted incidence or prevalence and the standard error will be recalculated based on the numerator and denominator values presented in each study. Furthermore, the prevalence or incidence may be reported using the direct method of standardization, adjusted for the variables of age, study location, and presence of co-infection. If the study does not provide data for calculating adjusted incidence or prevalence, the researchers will request this information from the study authors.

Additionally, a meta-analysis will be performed using a random-effects model due to the potential heterogeneity among studies. The random-effects model is applied when the aim is to combine several studies that have similar objectives but are conducted in different ways (ie, exhibiting methodological heterogeneity) [25]. Moreover, the Freeman-Tukey double arcsine transformation will stabilize variances to maintain the estimates of individual effects of each study [26]. Cochran Q test will assess the heterogeneity among studies [27]. *I*^2^ values of 25%, 50%, and 75% will represent low, medium, and high heterogeneity, respectively [28].

Studies will undergo a subgroup analysis using clustering variables (eg, study location, study population, method of syphilis diagnosis, mean sample size, year of data collection, sampling methods, and methodological quality) to investigate possible sources of heterogeneity [20].

Analyzes will be performed using the Review Manager (RevMan) software (version 5.4; Cochrane Collaboration) and the R software (R Core Team), considering a 95% CI.

## Results

The protocol has been registered in PROSPERO (CRD42022329329). The screening of the studies in the databases has already started, and the entire systematic review is expected to be completed by December 2023. The results of the study will provide evidence that can support decision-making regarding strategies to control syphilis in Brazil and countries with similar health, demographic, and socioeconomic profiles. Results will be disseminated through publication in peer-reviewed journals and presentation at scientific events.

## Discussion

### Expected Results and Practical Implications

After a preliminary search, we found studies in Brazilian cities that analyzed epidemiological data and predictors of syphilis in pregnancy and congenital syphilis [13-17,29]. However, no study has organized and summarized data to perform a broader analysis of this public health issue.

Summarizing local studies will allow the analysis and discussion of epidemiology and risk or associated factors of syphilis in pregnancy and congenital syphilis, considering sociocultural, demographic, spatial-temporal, economic, and sanitary differences in each location. Thus, this systematic review will help in the decision-making of health care managers and professionals to control these diseases in Brazil according to location heterogeneity.

### Limitations

Some limitations that may compromise the quality of evidence can be found in the systematic review, such as heterogeneity among studies, wide CIs, and uncertainty of estimated effects.

## Abbreviations

GRADE: Grading of Recommendations, Assessment, Development, and Evaluations
PRISMA-P: Preferred Reporting Items for Systematic Review and Meta-Analysis Protocols

## Data Availability

All data generated and analyzed in this study are available upon request from the corresponding author.
